# Production of new functional coconut milk kefir with blueberry extract and microalgae: the comparison of the prebiotic potentials on lactic acid bacteria of kefir grain and biochemical characteristics

**DOI:** 10.1007/s13197-024-05974-6

**Published:** 2024-04-05

**Authors:** Doğan Kürşad Aktas, Sevcan Aydin

**Affiliations:** Division of Biotechnology, Biology Department, Faculty of Science, Istanbul University, Vezneciler, 34134 Istanbul, Turkey

**Keywords:** Blueberry extract, Microalgae, Prebiotic activity, Probiotics, Lactic acid bacteria, Nanopore-based DNA sequencing

## Abstract

Probiotic foods are recognized for their importance on human health. Kefir is a versatile probiotic food that can be made from non-dairy sources for vegan diet. This study evaluated the addition of microalga *Haematococcus pluvialis* (0.50% w/v) and blueberry *Vaccinium myrtillus* (0.50% w/v) extracts to compare their influence on the biochemical properties and the bacterial community of coconut milk kefir through Nanopore-based DNA sequencing. Results revealed that the *V. myrtillus* increased the microbial diversity in coconut milk kefir with more abundant Proteobacteria species such as *Lacticaseibacillus paracasei* (22%) and *Lactococcus lactis* (6.3%). Microalga demonstrated the opposite effect on C, making Firmicutes represent the whole of the microbiota. Biochemical analysis revealed increased fat content in the kefir samples, with the C1 registering 1.62% and the 1.07% in C2, in contrast to the control group’s 0.87% fat content. The crude protein content exhibited a decrease in both samples compared to the control group (0.00% and 0.88% versus 1.07%). These findings suggest that fortifying vegan kefir with prebiotics has the potential to induce significant alterations in the kefir microbiota.

**Graphical abstract:**

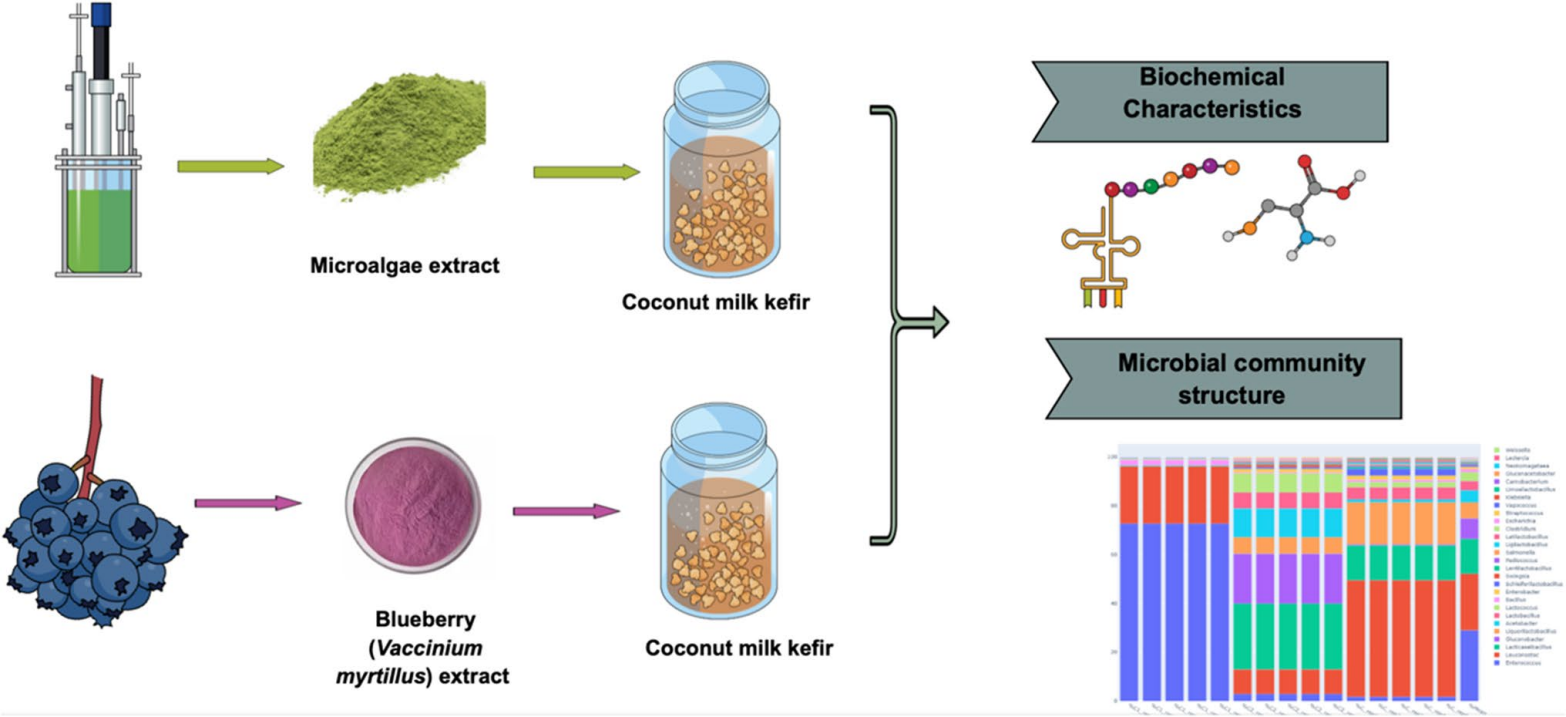

## Introduction

The gut is a bioreactor with diverse and complex microbes. The composition of the microbial flora in the human intestine is acknowledged as an important part of human health. Studies have focused on modifying the gut microbiota with probiotics for decades. Probiotics are the inclusion of beneficial bacteria to the human diet in aim to modulate the microbiota of the intestines (Thursby & Juge 2017). Due to its probiotic properties, kefir is the focus of scientific studies. As a traditional beverage that’s made up of beneficial bacteria which can alter the gut microbiota and help the consumer’s health, kefir is a viable way to boost the nutritional content of an individual’s diet (Peluzio et al. 2021). Plant-based milks from water-soluble extracts can develop new functional foods (Chen et al. 2018). An interesting option among water-soluble plants is water-soluble coconut extract, which is obtained from the endosperm of the coconut. Notable physical characteristics of coconut milk are a white color that’s similar to milk and an aroma that’s pleasurable to its consumer, which is important because sensory quality is a critical determinant to the consumption of a beverage. On the other hand, coconut milk can help develop central nervous system function as well as help sustain immune system (Sethi et al. 2016). Coconut milk curd consists of 1.1 g protein per 100 g of curd, making it a suitable dietary recommendation for lactose-intolerant people (Neelaveni et al. 2023). This property of coconut milk also makes it a promising substrate to kefir grains, which is known to colonize milk by conducting orderly changes on the metabolite content (Blasche et al. 2021). Coconut milk fermentation adds probiotics, boosts antibiotic activity, and enhances its anti-cancer potential (Ts et al. 2017). Coconut milk in kefir preparation has been optimized, and its nutritional and biochemical traits were studied. Additionally, studies have explored potential health benefits associated with the metabolites present in the prepared kefir (Mauro & Garcia 2019).

Prebiotics are non-digestible food ingredients that are degraded by the gut microbiota, stimulating the growth and activity of beneficial microorganisms in the gut. Prebiotics can boost the health benefits of kefir, a probiotic milk product. (Al-Sheraji et al. 2013). Recent studies aimed to evaluate the biochemical and microbial structure of fermented food. One experiment tested the persistence of beneficial bacteria by experimenting with samples with and without the addition of prebiotics. The results showed that prebiotic addition has enabled kefir bacteria to persist over time in fecal communities. Moreover, the addition of prebiotics has increased the relative abundance of *Bifidobacterium sp*., which most likely originated from host’s gut microbiota (Christensen et al. 2022). On the other hand, another experiment evaluated the effect of the addition of *Spirulina platensis* in vegan kefir. *Spirulina platensis* increased lactobacilli and lactococci in soy and almond milk kefir for 21 days. The highest counts of lactobacilli and lactococci were found in the soymilk kefir samples with 0.50% *Spirulina platensis*, which reached 8.48 log CFU/ml and 8.22 log CFU/ml, respectively (Sözeri Atik et al. 2021). The results indicate that prebiotics enhance the presence of beneficial bacteria species in fermented food, especially in kefir. It’s possible to suggest that the addition of prebiotics to kefir may produce a functional food with enhanced health benefits.

Blueberry (*Vaccinium myrtillus*) contains anthocyanins in high concentrations, thus show immense antioxidant properties. Blueberries can modify the gut microbiota to reduce obesity-induced inflammation and prevent pathogen infections (Zhou et al. 2020). In addition, blueberries are a source of fermented fibers that pose beneficial effects on the probiotic microorganisms and their metabolisms. Consumption of beverages produced with a wild blueberry powder increased the biomass of *Bifidobacterium spp*. in rodents, while dietary supplementation with whole blueberries changed the microbiota’s makeup. Recent studies added various prebiotics to kefir and assessed their effects on the kefir microbiota. The prebiotic effects of the addition maintained a viable population of bacteria and yeasts during refrigerated storage (Mendes et al. 2021). Inulin added to soymilk fermented with kefir grains increased the survival and sensory quality of *Lactobacillus*, *Lactococcus* and yeast (Santos et al. 2019). Studies examining other prebiotics have evaluated the effects of supplements on both microbiota structure and biochemical properties. On the other hand, there hasn’t been any studies on blueberry powder’s effect and potential on the probiotic bacteria mass and activity.

Kefir-based foods and beverages are biotechnologically important, but microalgal biomass addition is understudied. There are only a limited number of studies on effects of microalgae biomass as a high-quality food supplement to enhance the nutritional and bioactive properties of kefir fermentation. Microalgal biomass may be a potential milk substitute for kefir grains due to its carbohydrate content and bioactive molecules (Atik et al. 2021; Nascimento et al. 2022). This study analyzes coconut milk kefir microbiota and tests the effects of adding microalgae (0.50% w/v) and blueberry extract (0.50% w/v). Nanopore-based DNA sequencing helps us understand how beneficial bacteria and potential prebiotics (microalgae and blueberry) affect plant-based fermentation.

## Material and methods

### Preparation of kefir

The sample of *H. pluvialis* SCAAP 34/7 was provided by Scottish Marine Institute in Scotland, United Kingdom. and produced in photobioreactor using Bold Basal Medium (3N-BBM + V). Microalgae were centrifuged at 5000 rpm for 10 min following incubation. After being cleaned with distilled water, the resultant mass was dried in a freeze drier. After being reduced to powder in a mortar, the bulk was stored at –20 °C for future investigation.

Vegan kefir starter culture and freeze-dried blueberries was purchased from a local distributor.

Three kefir samples were produced from coconut milk (500 ml each) inoculated with plant-based culture (4 g: 500 ml) 21. C had no additives, C1 had 5 g (%0.5) of lyophilized microalgae, and C2 had 5 g (%0.5) of blueberry extract. Fermentation of the samples was conducted in an oven set to a temperature of 25 °C for and left for a fermentation period of 24 h.

### Genetic analysis of coconut milk kefir

Standardized workflows were used for metagenomic analysis targeted to 16S rRNA (Cusco et al. 2018). The primer pair for amplicon libraries targets a 1400 bp zone spanning the V1-V9 region of the gene (Zeng et al. 2013; Klindworth et al. 2013). The resulting Oxford Nanopore Technologies Nanopore barcode DNA sequences are added to the target specific primer pairs’ 5’ end. Target specific primer-connector sequences are TTTCTGTTGGTGCTGATATTGC—AGRGTTTGATYHTGGCTCAG -3’ for the forward primer and 5’- ACTTGCCTGTCGCTCTATCTTC—TACCTTGTTAYGACTT -3’ for the reverse primer. First PCR Proof Reading DNA Polymerase 2 × Reaction Mix and were used from each primer that’s 200 nm long. A thermal cycle program consisting of steps followed in the PCR process. The following thermal cycling program was followed in the PCR device: 3 min at 95 °C; 25 cycles at 95 °C for 30 s, at 55 °C for 30 s, and at 72 °C for 90 s; 5 min at 72 °C. The PCR product was run on an agarose gel to confirm its size (~ 1450 bp) and purified using the PCR Product Purification Kit.

The ligation sequencing kit 1D (SQK-LSK108; Oxford Nanopore Technologies) was used to make the amplicon library and update the MinION device. A positive control of 5 l of lambda phage DNA was added to the 45 l of barcoded DNA mix containing 1–1.5 g of DNA. DNA end repair and dA splicing were carried out using the New England Biolabs NEBNext End Repair/dA-tailing Module kit. Beckman Coulter’s Agencourt AMPure XP beads kit was used for purification.

For adapter ligation, 0.2 pmol tips and 50 l of Blunt/TA ligase master mix (New England Biolabs) with 20 l of adapter mixture were incubated at room temperature for 10 min. DNA library sequencing mixture (14 l), loading beads (25.5 l), and running buffer mixture (35.5 l). After priming and prepping the R9.4 flow cell, the sequencing mixture was loaded. Using MinION*™* control software, MinKNOW*™* version 0.46.1.9 (Oxford Nanopore Technologies—ONT, Oxford, UK), a 48-h (R9.4) sequencing experiment was carried out (R9.4). Data readings were gathered using the software Metrichor*™* agent and workflow 1.2.2 rev 1.5. (Version 0.16.37960). GuPPy v3.1.5 software (https://hpc.ilri.cgiar.org/guppy-software) converted the sequencing findings from fast5 to fastq via base-calling and de-multiplexing. Porechop v0.2.3 software (https://github.com/rrwick/Porechop) was used to purify barcode and adaptor sequencing. On both sides of the sequences, 45 bases were cleaned off, eliminating universal primers and tags in the process. Following sequence cleaning, the readings between 1350 and 1550 bp in length were filtered, and the remaining readings were disregarded for analysis.

With the help of a customized process and the platform mothur v.1.39.5, cleaned readings were evaluated. Using the similarity matrix to calculate the distances between readings with a similarity of more than 99%, sequences were deconstructed, aligned, and OTUs were created. The produced OTUs were compared to the RDP 16S rRNA database to conduct the taxonomic annotations. Associating OTUs that were identified as belonging to the same genus produced statistical findings. In order to visualize the organisms that were matched with the OTUs, the quantitative results, and the specimen-specific metadata, Mintitab and R software were used. Results are represented with a dendrogram of the samples’ proximity based on their diversity and amount. “Pearson” similarity method and Single-linkage hierarchy clustering method were used for proximity calculation. One of the examples is indicated as “mean”. This sample represents the average variation of all Grasses in percentage. The samples on the horizontal and vertical axis of the graph are colored so that the similarity ratio with the other samples is between 0 and 1. The higher the similarity ratio, the darker the color, thus approaching 1, on the contrary, the samples diverge from each other. All taxonomic levels of the samples are considered while performing this analysis.

### Physicochemical and biochemical analyses

The pH values of the control and the fortified samples were measured by a pH meter (NMKL 179, 2005). TS 1330 standards were used in determining the total titratable acidity values of the samples, which were expressed in g/100 mL. Protein content was analyzed by Dumas method (ISO/TS 16634–2:2009) with Thermo scientific flash EA1112 CHNS-O elemental analyzer. The calculation’s output format is “% Crude Protein.”

NMKL 160 Gravimetric Method was used in analyzing the total lipid content of the kefir samples. In order to release bound fat and transform fatty acid salts into free acids, kefir samples were boiled in diluted hydrochloric acid. Before filtering and drying, the residue was extracted with hexane. The mass of the residue was determined after distilling the solvent off the mass. “%” lipid” was used as the expression of the fat content of the samples.

High performance liquid chromatography (HPLC) was employed for sugar content analysis in C, C1, and C2, utilizing a Shodex Asahipak NH2P-50 4E column (4.6 × 250 mm; Showa Denko KK, Tokyo, Japan) (Jalaludin and Kim 2021). Water and acetonitrile were utilized as the mobile phase (75:25, v/v) during seperation, which took place at 30 °C. In the running time of 20 min, the injection volume was of 20 μL and system flow was 1.1 mL/min. A refractive index detector (RID 10A) was used in detecting the sugar in kefir samples and “% dry weight was the format of the lactose amounts of the samples.

A gas chromatograph (Agilent 8890-5977B GC–MS and Agilent 19091S-433UI HP-5 ms ultra inert fused silica capillary column, 30 m × 0.25 μm × 0.25 μm) separated the volatile-organic compounds. A temperature program (1 min at 40 °C, raised to 240 °C at 10 °C/min, then 1 min at 240 °C) had a total runtime of 27.1 min. Injector was used in the splitless mode, with a 1 μL of injection volume and a total flow of 16 mL/min. As the carrier gas, Helium gas with a 1.0 mL/min constant flow rate and 7 psi of column head pressure was used. Ion source had 70 eV of ionizing energy and a temperature of 230 °C. The National Institute of Standards and Technology (NIST) reference library identified the VOCs, compared the retention times (Rt) and mass spectra of authentic standards.

Agilent 8890-5977B GC–MS and Agilent 19091S-433UI HP-5 ms ultra inert fused silica capillary column (30 m × 0.25 μm × 0.25 μm) was used in analyzing of the fatty acid profile. A temperature program consisting of the steps (1) 1 min at 100 °C; (2) for 1 min, an increase to 180 °C at 30 °C/min; (3) for 1 min, an increase to 220 °C at 2 °C/min; (4) for 7 min, an increase to 270 °C at 30 °C/min. The runtime of this temperature program was 34.3 min. Injection was used in splitless mode with an injection volume of 1 μL and a total flow of 19 mL/min. As the carrier gas, with 1.0 mL/min of constant flow rate and 7 psi of column head pressure, Helium was used. MS scanning was conducted with the parameters: normal scanning mode, 650 min of run time, and scanning from m/z 40–600. 70 eV was set as the electron energy and 230 °C was set as the source temperature. The National Institute of Standards and Technology (NIST) reference library was used in determining the identification of the VOCs, the retention times (Rt) comparison and authentic standards’ mass spectra.

### Nucleotide sequence accession numbers

The Oxford Nanopore sequences are available through the National Center for Biotechnology Information (NCBI) Sequence Read Archive (https://www.ncbi.nlm.nih.gov/biosample/33229168) under project SAMN33229168.

## Results and discussions

### Influencing of microalgae and blueberry extract coconut milk kefir fermentation biochemical characteristics

Novel prebiotics alter the chemical composition of fermented food products. Lactic acid bacteria consume carbohydrates and produce metabolites that cause this change (Costa et al. 2018). To better assess this change, pH and titratable acidity are used as two important metrics that define the acidity of the functional food. In the results of our measurements, the pH values control group (C) and coconut milk kefir with microalgae addition (C1) were 4.93 and 4.95, respectively, while coconut milk kefir with blueberry addition (C2) is slightly more acidic at a pH value of 4.76. This difference can be attributed to the C2 having a more diverse microbiota that consisted of more abundant lactic acid and acetic acid bacteria. LABs are known to produce organic acids that affect the pH value in the environment, causing their environment to get nearer to the more acidic side of the scale (Nikolaou et al. 2019). Coconut milk kefir with *H. pluvialis* microalgae has a higher pH value due to astaxanthin, a carotenoid that protects cells from oxidative stress (Sözeri Atik et al. 2021).

Protein determination in samples is shown as percentages. C consisted of 1.07% of crude protein while C2 had a lower protein determination profile of 0.88%. Though, there has not been any protein determination detected in coconut milk kefir with microalgae addition. Amino acids are the building blocks of proteins and are involved in various biological functions. Lysine, methionine, and tryptophan are essential amino acids from the diet. Vegan diets may lack some of these essential amino acids, especially lysine, which is mainly found in animal products (Hou et al. 2015). Therefore, coconut milk kefir may provide a valuable source of amino acids for vegans. Fat content varied greatly in the biochemical assessment. The coconut milk kefir sample had 0.87% fat content, while microalgae addition almost doubled this proportion to 1.62%. Blueberry addition has also increased the fat proportion over the control group with 1.07% fat content.

Table 1 shows the fatty acid profiles of the samples by percentage. The most abundant fatty acid in our samples was lauric acid, which consisted of almost half of the fatty acid profile in C (45.33%) and C2 with blueberry addition (43.18%). In stark contrast, no lauric acid has been detected in C1 with microalgae. Linoleic acid has been detected in all samples, though the percentage of linoleic acid was higher in the control group and kefir with blueberry addition (30.31% and 32.19%, respectively), than the kefir sample with microalgae addition (24.02%). Pentadecanoic acid hasn’t been detected in coconut milk kefir, while the sample with microalgae addition had three times the value (0.12%) when compared to C2 (0.04%). The outliers in these results are 7, 10, 13-Eicosatetraenoic acid and cis-11, 14-Eicosadienoic acid, which appeared only in coconut milk kefir fortified with *H. pluvialis*.

**Table 1:** The percentage of fatty acids profile for kefir samples (C, C1, C2)

Analytes	Synonym	Sample C	Sample C1	Sample C2
Decanoic acid	Capric acid	2,983	2,927	2,147
Dodecanoic acid	Lauric acid	11,387^a^	10,302	8,239
Tetradecanoic acid	Myristic acid(C14:0)	5,80	5,69	3,64
Pentadecanoic acid	C15:0	–^b^	0,12	0,043
Palmitoleic acid	C16:1w7	0,05	1,3	0,067
Hexadecanoic acid	Palmitic Acid (C16:0)	10,965	14,883	11,556
Heptadecanoic acid	C17:0	0,08	0,046	0,027
γ-Linolenic Acid	Gamma-Linolenic Acid (C18:3w6)	–	4,27	–
(Z,Z)-9,12-Octadecadienoic acid	Linoleic Acid (C18:2w6)	30,31	24,028	32,195
Oleic acid	Oleic Acid (C18:1w9)	11,463	11,037	18,996
Elaidic acid	trans-9-Octadecenoic acid	0,496	0,513	0,628
Octadecanoic acid	Stearic Acid (C18:0)	3,27	1,94	3,153
7,10,13-Eicosatrienoic acid	C20:3n-7	–	0,092	–
cis-11,14-Eicosadienoic acid	C20:2	–	0,037	–
cis-13-Eicosenoic acid	–	0,031		0,029
Eicosanoic acid	Arachidic acid (C20:0)	0,116	0,05	0,12
Heneicosanoic acid	C21:0	0,03		
Docosanoic acid	Behenic acid (C22:0)	0,429	0,106	0,375
Tricosanoic acid	C23:0		0,058	
Tetracosanoic acid	Lignoceric acid (C24:0)	0,095		0,069
Omega 6	–	0,13	0,02^c^	0,00^d^
Saturated Fatty Acids	–	0,78	0,90	0,80
Polyunsaturated Fatty Acids	–	0,13	0,02	0,00
Monounsaturated Fatty Acids	–	0,71	0,11	0,07
Unsaturated Fatty Acids	–	0,84	0,13	0,07
Eicosenoic acid (C20:1)	–	–	1,12	–
Linoleic acid (C18:2)	–	0,40	6,86	1,57
Stearic acid (C18:0)	–	3,83	16,24	3,77
Palmitic acid (C16:0)	–	10,85	31,25	10,26
Myristic acid (C14:0)	–	17,72	–	16,62
Caprylic acid (C8:0)	–	7,80	–	7,70
Caproic acid (C6:0)	–	0,70	–	0,62

In our measurements, the control group did not present any Pentadecanoic acid (C15:0). The rates were 0.12% in C1 and 0.043% in C2. Linoleic acid percentage was 30.31% in coconut milk kefir, 32.20% with blueberry, and 24.03% with *H. pluvialis*. The rate of Omega 6 was 0.13% in C. 0.02% in C1 while none detected in C1. 11.46% of the fatty acid profile of the control group consisted of oleic acid, while C1 had shown a rate of 11.04% and C2 at 19%. On the other hand, Gamma-Linolenic acid (4.27%), 7,10,13-Eicosatrienoic acid (0.09%) and cis-11, 14-Eicosadienoic acid (0.037%) were the several fatty acids that were only detected in C1.

The samples contain fatty acids such as lauric acid, linoleic acid, oleic acid, and gamma-linolenic acid. These compounds have health benefits such as antimicrobial, anti-inflammatory, antioxidant, and neuroprotective effects (Xing et al. 2023). Some of them, such as linoleic acid and gamma-linolenic acid, are also considered essential fatty acids, so they cannot be synthesized by the human body, therefore must be obtained from the diet (Das 2006). Vegan diets may lack some of these essential fatty acids, therefore, the product may provide a valuable source of fatty acids for vegans.

### Effects of microalgae and blueberry extract on the abundance and diversity of microbial community structure of coconut milk kefir

**Fig. 1:**
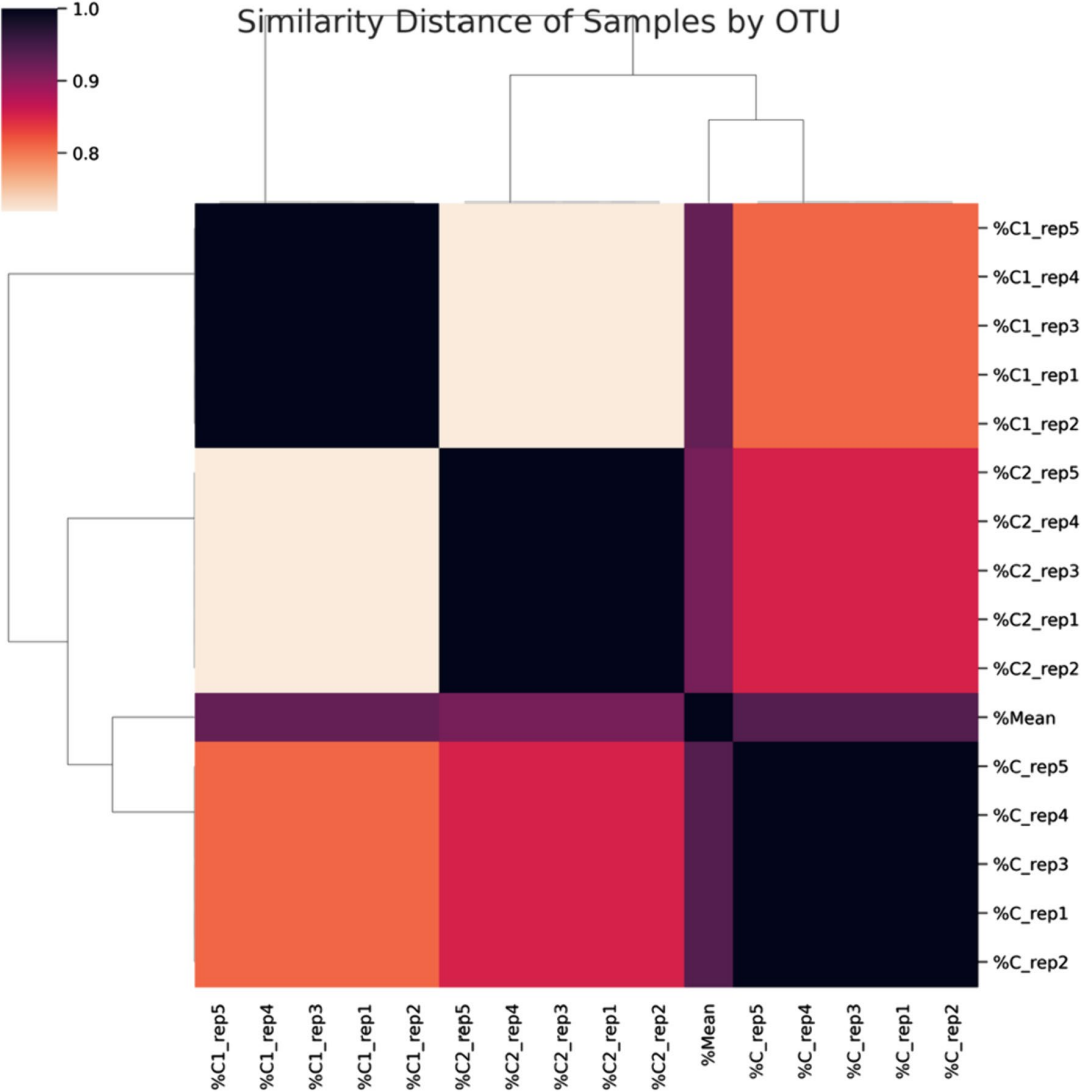
Pearson correlation analysis of C, C1 and C2 kefir samples

To assess the bacterial abundance and diversity in coconut milk kefir, three samples (C, C1, and C2) were subjected to similarity, alpha, and beta diversity analyses. Changes made to the microbiota profiles of samples by the addition of prebiotics are presented as Dendrogram as can be seen in Fig. 1. While the similarity distance is close between C1 and C2, both microbiotas’ similarity distances appear more distant to the control group. The abundance of the different bacterial taxa is presented as operational taxonomic unit (OTU) numbers, which h is 2425, 4279 and 4636 for C, C1 and C2, respectively. The diversity change may result from the prebiotic sources’ impact on kefir microbiota (Blasche et al. 2021).

**Fig. 2:**
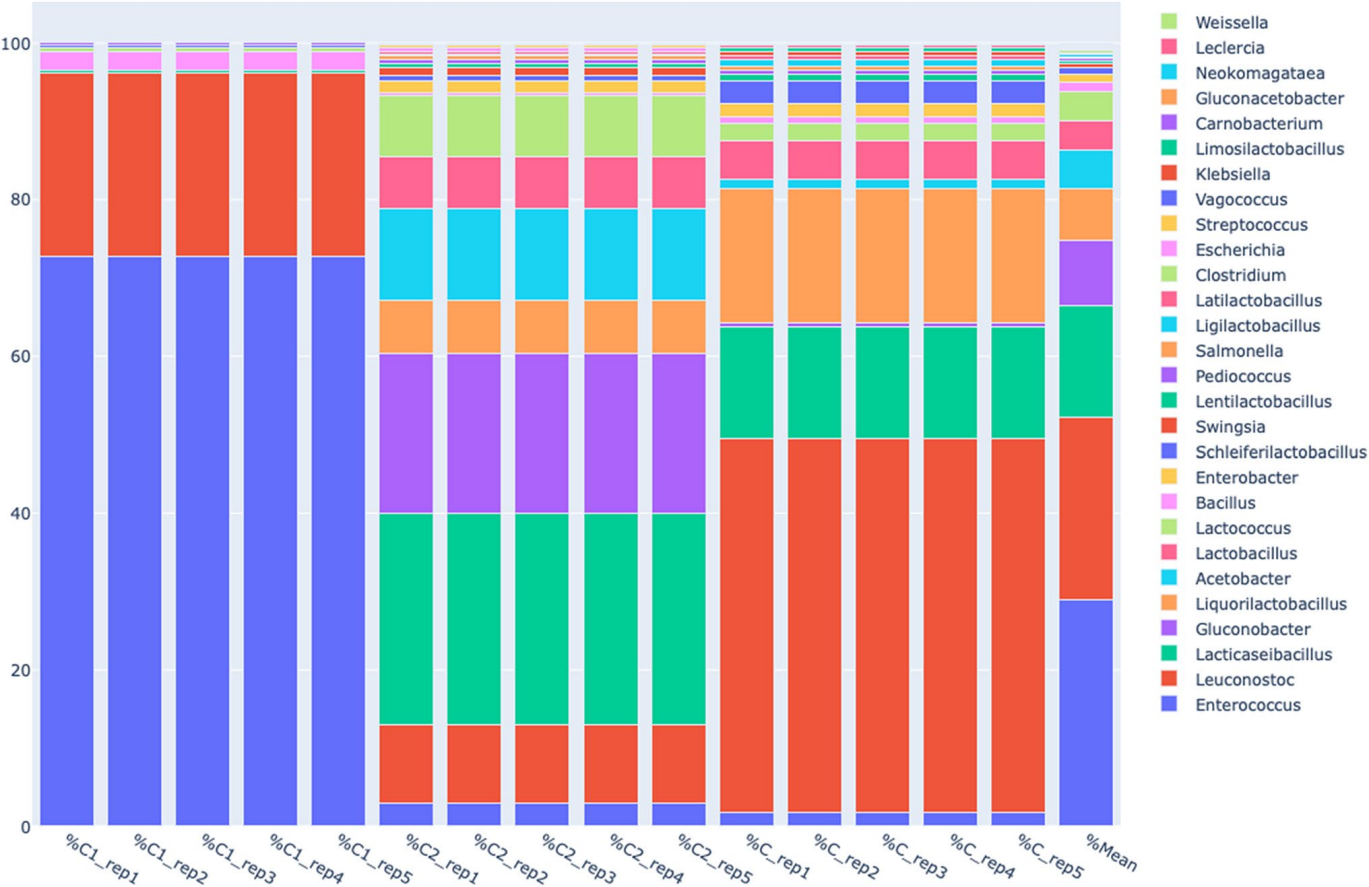
Qiime Barplot percentage display of microbial community diversity and quantity graphs at genus level

Relative abundances of phyla in samples can be seen in Fig. 2. There are two phyla observed in all samples, Firmicutes and Proteobacteria. In C, Firmicutes phylum represents 95.5% of the microbiota while the remaining 4.4% consists of Proteobacteria alone. This finding is parallel to the results found in studies examining samples from traditional and commercial kefirs (Biçer et al. 2021). Interestingly, C1 microbiota was under complete dominance of phylum Firmicutes in our study. However, C2 had more Proteobacteria, representing 35.85% of the microbiota. 4 different classes under these two phyla were detected in the samples: Bacilli, Alphaproteobacteria, Gammaproteobacteria and Clostridia, by the order of abundance. While Bacilli was abundant in all samples, Alphaproteobacteria and Gammaproteobacteria presence increased only in C2. On the other hand, C1 only had Clostridia. In the level of ordines, results show that Lactobacillales made up 94.7% of the C microbiota, and 97.1% of C1. Blueberry addition resulted in a more diverse microbiota in C2, as Lactobacillales represented 63.7% of the microbiota.

**Fig. 3:**
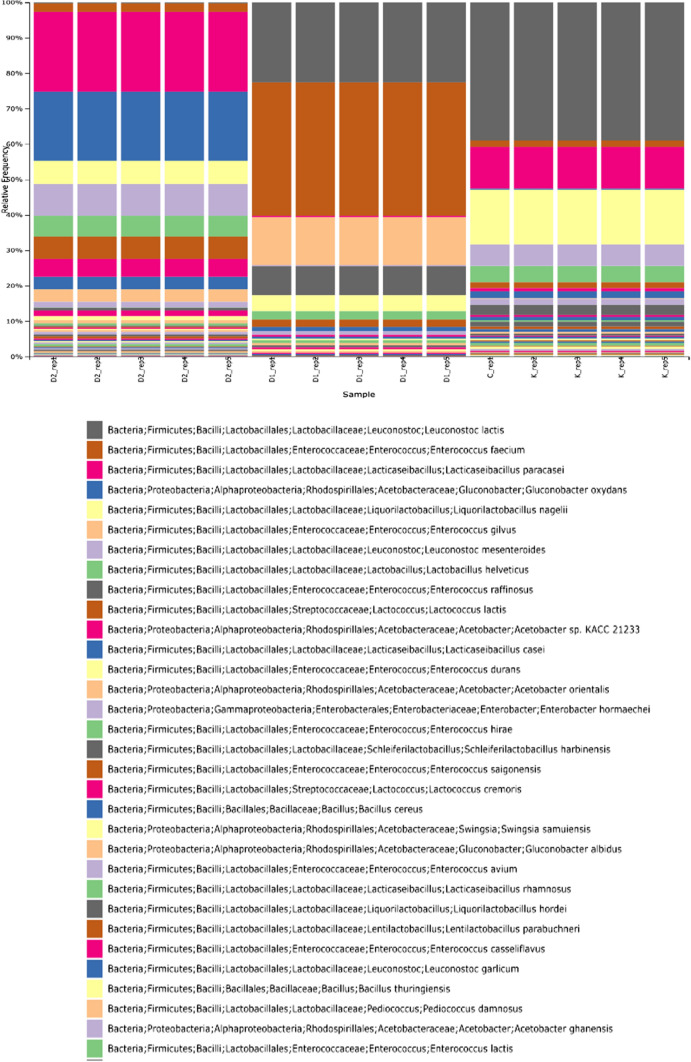
Bacterial community structure at C, C1, C2 species level

At the genus level, all three samples have shown a total of 28 different genera in their microbiota, as can be seen in Fig. 3. In C, *Leuconostoc* is the most abundant genus by 47.75%. C1 showed much less diversity, with over 95% of its microbiota consisting of *Enterococcus* and *Leuconostoc*.

Upon closer look to C2 at genus level, the more abundant level of *Lactobacillus* and *Lactococcus* in the microbiota becomes apparent, representing 6.49% and 7.86% of the whole microbiota, respectively. These genera possess health benefits such as immunomodulation and protection from pathogens (Slattery et al. 2019). Though, *Gluconobacter* is the most abundant genus in this sample, making up %20,44 of the kefir microbiota alone. *Gluconobacter oxydans* is a notable species under this genus, which is found to offer probiotic properties, adding to the number of the beneficial bacteria species in this sample (Begum et al. 2015). Another notable species with an increase in its abundance in C2 is *Lactobacillus paracasei*, which makes up 22% of the total microflora. This increase is up from 11% in C and 0.44% in C1. *Lb. paracasei* has health benefits such as antioxidant, immunomodulatory, antiproliferative, and lipid-improving effects (Bengoa et al. 2021; Karaffová et al. 2021). A similar species in terms of health benefits is *Leuconostoc mesenteroides*, which was detected in all samples, but shown more prevalence in C2. *Leunocostoc mesenteroides* has been reported by Chang-Liao et al. (2020) of its ability to help mitigate a type of viral diarrhea. *Leunocostoc* mesenteroides also produce dextransucrase, isolable from water kefir (Miljkovic et al. 2017).

### Comparison of microalgae and blueberry effects on the prebiotic potential of coconut milk kefir

Microalgae is a functional food additive that modulates human gut microbiota and acts as a prebiotic (Hernández et al. 2022). The effect of microalgae and blueberry extracts that is added as probiotics to the kefir samples made with coconut milk caused a significant difference in the kefir microbiota compared to the control group. *Haematococcus pluvialis* present in coconut milk kefir sample is proven to have antioxidative response when added into powder or flour of different pastries (Mofasser Hossain et al. 2017). Moreover, *H. pluvialis’* ability to produce astaxanthin bears benefits such as anti-inflammatory effects, modulation of immune system and improvable of cardiovascular health (Chan et al. 2019). Though, in comparison to the control group, the addition of *H. pluvialis* also introduced adverse effects against beneficial species in the kefir flora, such as *Lacticaseibacillus paracasei*, *Lactobacillus helveticus* and *Gluconobacter oxydans*; which possess antioxidative properties and immunomodulatory activity (Bengoa et al. 2021). Thus, microalgae in coconut milk kefir fermentation made C1’s microbiota less diverse and also free of Proteobacteria.

The antioxidant capability of blueberry species *Vaccinium myrtillus* is well studied (Zhou et al. 2020). In conducted studies, blueberry is also found to be capable of modulating specific gut bacteria in colon (Ntemiri et al. 2020). By comparison to the C and C1 with a much less diverse microbiota, it is notable that species under phylum Proteobacteria that is in the microbiota of the C2 has benefitted from blueberry’s influence on coconut milk kefir fermentation. In our experiment, *Lacticaseibacillus paracasei* had an OTU number of 285 in C and 1048 OTU in C2, showing 3.67 times more abundance. The findings of (Bengoa et al. 2021) reflect ours in this aspect. Kefir with blueberry addition also showed a boosting effect on the OTU number of *Vaccinium myrtillus* in coconut milk kefir fermentation*. Lactococcus lactis* is yet another species that thrived under the fermentation conditions in kefir with blueberry addition. Strains of *L. lactis* isolated from milk kefir have been reported to have more probiotic properties than those from raw milk isolated from kefir grains. Anthocyanin addition to the kefir yielded similar results, bringing diversity to an otherwise nondiverse microbiota dominated by *Lactobacillus* in kefir samples in the experiment. Anthocyanin also reduced pathogenic bacteria and increased *Lactobacillus* in kefir, matching recent findings (Aydin et al. 2022).

**Fig. 4:**
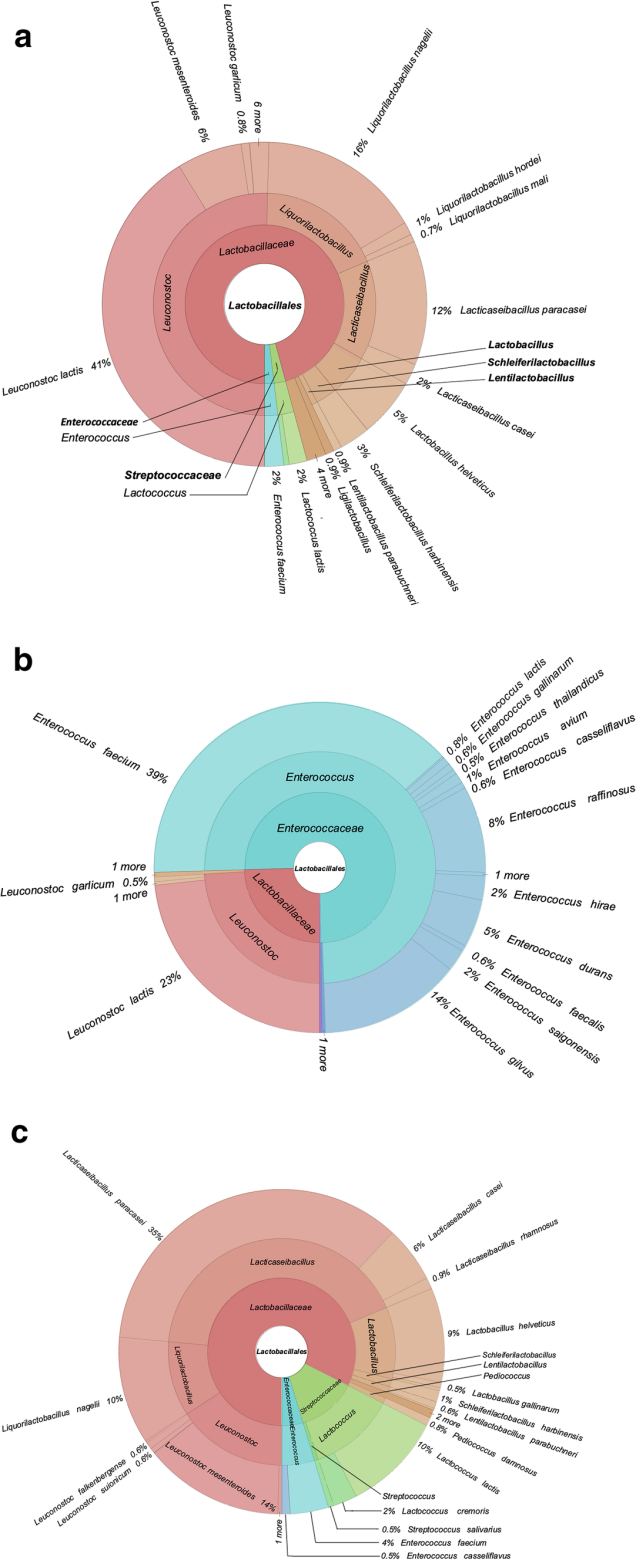
Abundance of *Lactobacillales* in coconut milk kefir from **a** C, **b** C1 and **c** C2

Figure 4 shows the relative abundances of bacteria under the order Lactobacillales in all three of the samples. Lactobacillaceae dominated C (96%) and C2 (82%) but was lowest in C1 (25%). Coconut milk kefir fermentation with *Haematococcus pluvialis* addition significantly changed the microbiota profile to be Enterococcaceae dominated (75%) with Enterococcaceae abundance among Lactobacillales shrinking to 24%.

Family Streptococcaceae was present in minute abundance in C, consisting of genus *Lactococcus* entirely, which made up 2% of the order Lactobacillales present in the sample. C2 possessed a significantly higher relative abundance of Streptococcaceae (13%) while no amount detected in C1 Enterococcaceae was scarce in C (2%) and C2 (5%) but surged in C1 (75%) after *H. pluvialis* fermentation. Most predominant species in the samples belonging to this family was *Enterococcus faecium* in C2 (39%). On the other hand, *Liquorilactobacillus* was absent in C1 and reduced in C2 (11% vs 18%), indicating *H. pluvialis* harmed kefir microbiota diversity. Similarly, minimal amount of *Lactobacillus* abundance was detected in C while C1, once again, didn’t consist of any *Lactobacillus*. In a similar fashion to other genera, C2 had shown increased abundance of *Lactobacillus* (10%).

Compared to other genera, *Leuconostoc* showed a different trend between all samples. *Leuconostoc* consisted 50% of the Lactobacillales in C while occupying 24% of the *Lactobacillales* in the sample. C2 had the lowest OTU number of *Lactobacillus* (16%). One possible reason for higher counts of *Leuconostoc* sp*.* in C2 than C1 is that the blueberry extract contains phenolic compounds that have antimicrobial and antioxidant properties which could give *Leuconostoc* sp. a competitive advantage in the microbiota (Ji et al. 2013). Another possible reason is that the blueberry extract might have provided *Leuconostoc* sp. with carbon and nitrogen sources that stimulated its growth and enhanced its metabolic activity, thus increasing its population.

Furthermore, two species detected across all samples under this family are *Leuconostoc lactis* 41–23-0,4 and *Leuconostoc mesenteroides* 6–0,4–14, which responded to the fermentation differently.

## Conclusion

Kefir is a popular fermented beverage that has gained attention due to its positive health effects and unique sensory properties. In this study, the microbiota properties of coconut milk kefir fermented with microalgae and blueberry were compared. Different fermentation agents affect flavor and health benefits. This could lead to the development of new kefir-based products that promote gut health and overall well-being while also bringing different compositions, consistencies, and tastes to the variety of kefir products present in the market.

The results showed that *V. myrtillus* increased the microbial diversity in coconut milk kefir with a higher abundance of Proteobacteria species such as *Lacticaseibacillus paracasei* and *Lactococcus lactis*. On the other hand, microalgae had the opposite effect on coconut milk kefir with no Proteobacteria present in the sample, resulting in a complete domination of phylum Firmicutes in the microbiota of the beverage. Different prebiotics can alter vegan kefir’s microbes without affecting its biochemistry. This information could be useful for the development of new vegan kefir products with specific health benefits and desired microbial profiles. Additionally, understanding the flavor profiles of kefir fermented with microalgae and blueberry could have a significance. Further studies conducted in food biotechnology could leverage these flavor profiles to create unique and interesting kefir-based beverages.

## Data Availability

The datasets generated during and/or analyzed during the current study are available in (https://www.ncbi.nlm.nih.gov/biosample/33229168) under project SAMN33229168.
